# Renal Peripheral Neuroectodermal Tumor Presenting at Age 78: Case Report

**DOI:** 10.1100/tsw.2008.109

**Published:** 2008-08-31

**Authors:** Michelle E. Koski, Jason M. Tedesco, Peter E. Clark

**Affiliations:** ^1^ Department of Urology, Vanderbilt University Medical Center, Nashville, TN, USA; ^2^ Department of Pathology, Vanderbilt University Medical Center, Nashville, TN, USA

**Keywords:** peripheral neuroectodermal tumor, renal tumor, Ewing sarcoma, oncology, urology, CD99, vimentin, synaptophysin

## Abstract

Primitive neuroectodermal tumor (PNET) of the kidney is a rare and aggressive tumor, often presenting in advanced stages and progressing rapidly. Renal PNET (rPNET) may affect a wide age spectrum, but the majority of cases are in young adults. We present a case of advanced rPNET in a 78-year-old woman. To our knowledge, this is the most advanced age of presentation of this neoplasm to date. We report on her presentation and management, and discuss the current clinical management of this tumor.

## PRESENTATION

A 78-year-old female presented with syncope, respiratory failure, and 1 week of painless gross hematuria. Computed tomography (CT) of the chest revealed a saddle pulmonary embolus and a left renal mass. CT of the abdomen and pelvis were then obtained, showing an enlarged left kidney with delayed function and an 8.0- × 6.4-cm heterogenous renal mass, with a retroaortic renal vein with venous involvement by the mass. There was no atrial mass on echocardiogram. There were no metastases on bone scan. Imaging studies were repeated after she was referred to our institution 1 month later for a second opinion and operative management. At that time, CT of abdomen and pelvis without intravenous contrast was obtained, showing enlargement of the mass to 10.9 × 10.3 cm with para-aortic lymphadenopathy and extension of the tumor thrombus to the level of an inferior vena cava (IVC) filter and possibly into the contralateral renal vein (Figs. 1 and 2). CT of the chest showed no metastases. Soon thereafter, she underwent left radical nephrectomy, retroperitoneal lymph node dissection, vena caval tumor thrombectomy, and inferior vena cava resection, the latter due to the finding of occlusion of the IVC at the level of the common iliac veins by bland thrombus at the time of surgery.

Gross pathologic evaluation revealed an 822-g radical nephrectomy specimen measuring 21.0 × 10.5 × 10.0 cm. A heterogenous tan-red mass with areas of hemorrhage and necrosis measuring 9.0 × 9.0 × 8.7 cm involved the majority of the renal parenchyma. Microscopic evaluation revealed a tumor with sheets of poorly differentiated, small round blue cells with scant cytoplasm and granular nuclei (Fig. 3). Immunohistochemistry showed strong expression of CD99 and vimentin, weak expression of synaptophysin and neuron specific enolase (NSE), and no expression of epithelial membrane antigen (EMA), AE1/AE3, CD45, CD56, or chromogranin (Fig. 4). On the basis of these findings, a diagnosis of

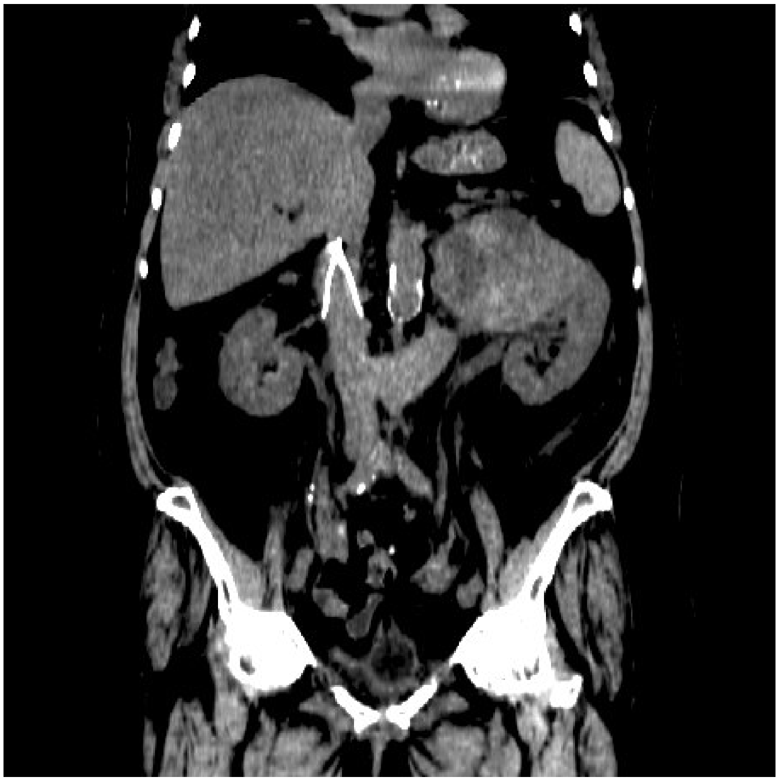

**FIGURE 1**. CT with coronal reconstruction demonstrating renal vein involvement.

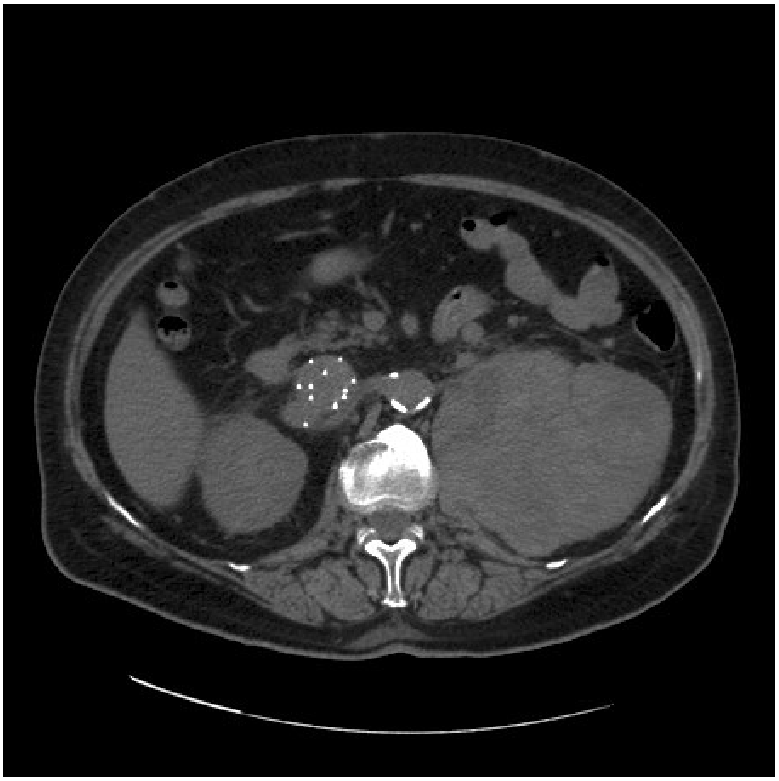

**FIGURE 2**. Transverse view of CT.

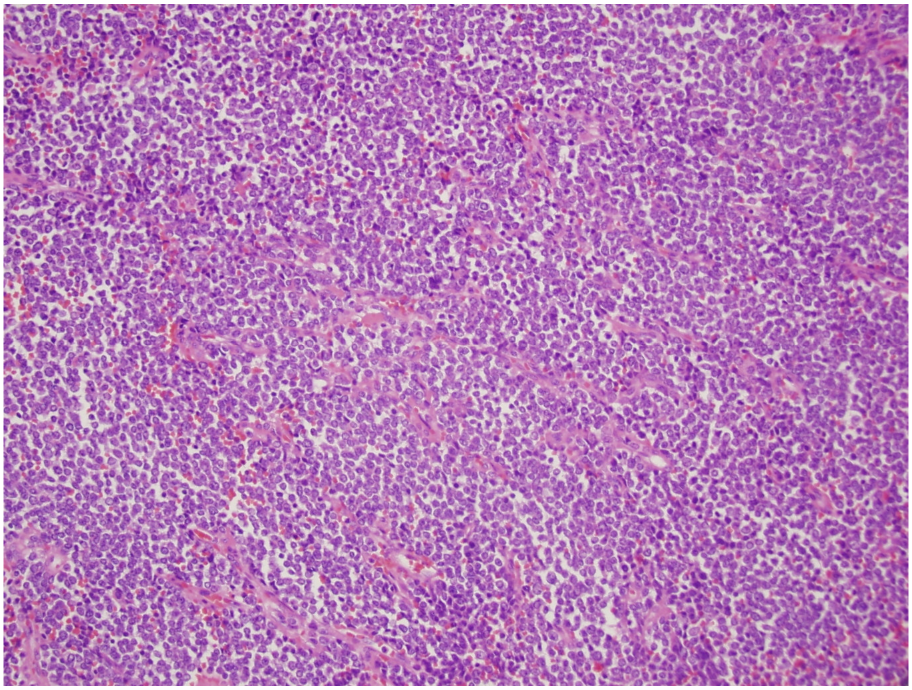

**FIGURE 3.** Low power view of tumor. Rosettes are not demonstrated in this specimen.

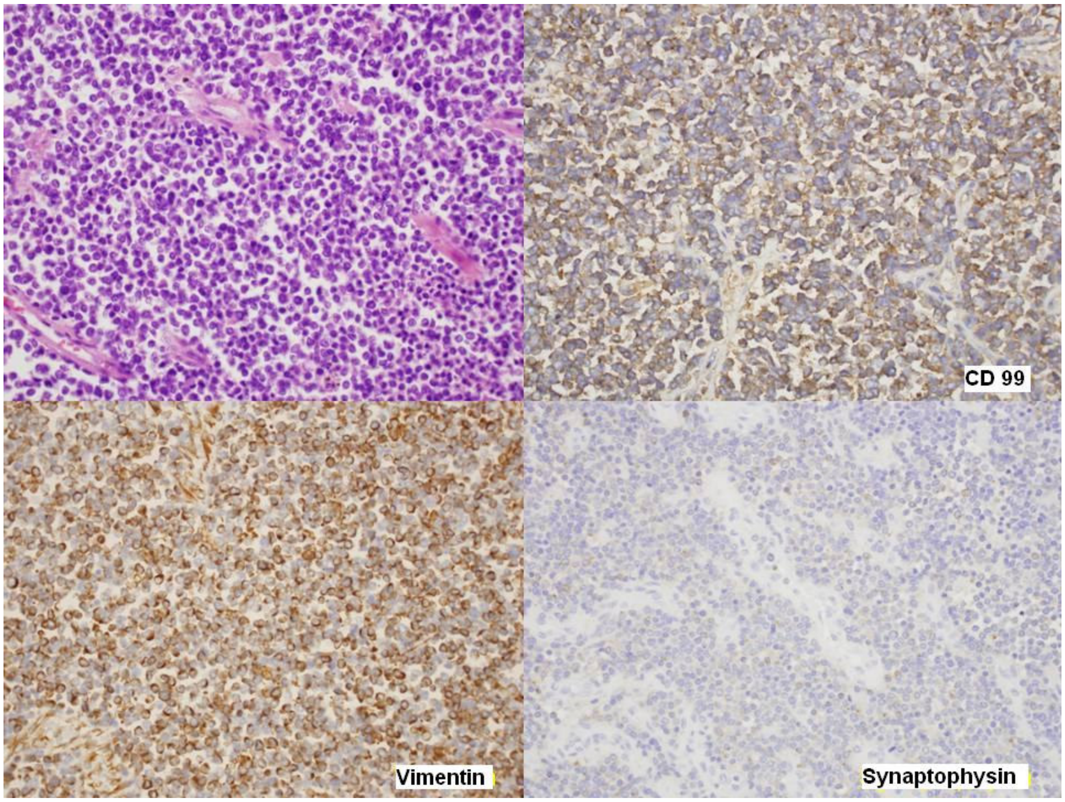

**FIGURE 4.** Immunohistochemical staining showing strong expression of CD99 and vimentin, and weak expression of synaptophysin. Upper left quadrant is untreated.

primitive neuroectodermal tumor (PNET) was rendered. The tumor focally extended beyond the renal capsule into the perirenal adipose tissue. All surgical margins were negative. Three hilar lymph nodes and five interaortocaval nodes were negative for tumor.

She tolerated surgery without complication and her postoperative course was essentially unremarkable. Two weeks later, she was admitted to an outside institution with dehydration and CT of the chest/abdomen/pelvis showed interval development of multifocal diffuse pulmonary nodules. She ultimately declined further therapy and was discharged to home hospice.

## DISCUSSION

PNET and Ewing sarcomas together comprise the Ewing family of tumors (EFT), a group of poorly differentiated, small round cell neoplasms primarily presenting as bone or soft tissue masses in children and young adults. PNET may arise from other peripheral locations and from a urologic standpoint, there have been infrequent reports of bladder, prostate, ureteral, and spermatic cord PNET[1,2,3,4]. Primary renal PNET (rPNET) is rare, with less than 100 reported cases in the literature. Although it may affect a wide age spectrum, ranging from 1 month to 72 years in one group (with a median age of 18 years), rPNET primarily affects young adults and behaves aggressively[5,6]. In a recent series of 52 published cases of rPNET, 57.6% of patients presented in advanced stages, with metastasis most frequently to lymph nodes, lung, and liver[7]. Another series demonstrated 5-year survival rates as low as 45–55%, despite aggressive treatment, with overall survival of 60 months in localized disease and 15 months in patients with nodal disease or distant metastases[6].

Our case illustrates the aggressive and rapidly progressive nature of this tumor, with marked growth of the primary over an interval of 1 month and development of pulmonary metastases 1 month after resection. Our patient is notable for an atypical age of 78 at presentation; this is the most advanced age at presentation that we have found in the literature. She had previously been in excellent health and had good functional status preoperatively.

The diagnosis of rPNET is challenging and often only determined by postoperative pathologic findings, given that patient presentation, laboratory studies, and imaging are often nonspecific. The "classic triad" of flank pain (85%), palpable mass (60%), and hematuria (37%) are common presenting symptoms[7]. Laboratory values are often normal, although cases of elevated LDH and NSE have been reported[7]. Radiographic findings of rPNET on CT, MRI, and ultrasound are largely nonspecific. Peripheral PNET (pPNET) typically appears on CT as an enhancing, heterogenous, soft tissue mass with cystic or necrotic areas and may variably show calcification[7,8]. Bright heterogenous signal intensity is noted on T2-weighted MRI[8]. It may appear hyperechoic or isoechoic on ultrasound[9].

Histopathological, immunohistochemical, and genetic analyses are concurrently employed to confirm the diagnosis. Microscopically, PNET is a poorly differentiated, round cell sarcoma with round to oval hyperchromatic nuclei and scant cytoplasm. Primitive neural features are cited, including rosette formation on the microscopic level and secretory granule formation at the ultrastructural level, although there can be atypical morphologies[5]. PNET expression of neural markers, including NSE, vimentin, S-100, neurofilament, synaptophysin, and chromogranin, can help to differentiate it from other EFTs[5]. PNET is almost universally positive for the cell surface glycoprotein MIC2 (CD99)[5]. Approximately 90% of Ewing sarcomas and peripheral PNETs express an EWS-FLI1 fusion protein that corresponds to a t(11;22)(q24:q12) chromosomal translocation[10,11]. Our diagnosis was confirmed by strong immunohistochemical presence of CD99 and vimentin, along with weak tumor cell expression of NSE and synaptophysin, and no expression of EMA, AE1/AE3, CD45, CD56, or chromogranin.

Standard therapy generally consists of systemic multiagent chemotherapy combined with surgery. Because rPNET is often diagnosed postoperatively on surgical pathology, chemotherapy is typically delivered adjuvantly[7]. pPNET is treated with chemotherapy regimens based on Ewing sarcoma regimens. Current chemotherapy regimens for Ewing sarcoma include doxorubicin, vincristine, cyclophosphamide, and actinomycin. An alternating course of ifosfamide and etoposide may be added[11]. Lastly, radiation therapy has been considered as an adjunct to surgery in cases of positive margins or invasion of Gerota's fascia, or in combination with chemotherapy in unresectable tumors, although its role in PNET is not well defined[6].

rPNET is rare, aggressive, and difficult to diagnose. Imaging and laboratory studies cannot definitively differentiate rPNET from other causes of renal mass. Young adults are more commonly affected, but this is not a fail-safe association, as demonstrated by our patient. Diagnosis is based on histology and immunohistochemistry complemented with cytogenetics. Pathologic evaluation can be challenging, even with these modalities. An aggressive combination of surgery and multiagent chemotherapy based on the treatment of other EFTs is recommended. Despite treatment, survival rates are low. It does appear that rPNET is being reported more and more frequently, possibly due to increased availability of immunohistochemistry and cytogenetic methods. Hopefully, the accumulation of cases and series in the literature will lead to collaborative efforts to develop more specific treatment protocols.

## ACKNOWLEDGMENTS

Special thanks to Joyce Johnson, M.D, who assisted in review of this article.
